# Genome rearrangement affects RNA virus adaptability on prostate cancer cells

**DOI:** 10.3389/fgene.2015.00121

**Published:** 2015-04-01

**Authors:** Kendra Pesko, Emily A. Voigt, Adam Swick, Valerie J. Morley, Collin Timm, John Yin, Paul E. Turner

**Affiliations:** ^1^Department of Ecology and Evolutionary Biology, Yale UniversityNew Haven, CT, USA; ^2^Department of Chemical and Biological Engineering, University of Wisconsin–MadisonMadison, WI, USA

**Keywords:** adaptation, evolution, genetic architecture, recombination, rhabdovirus, vesicular stomatitis virus

## Abstract

Gene order is often highly conserved within taxonomic groups, such that organisms with rearranged genomes tend to be less fit than wild type gene orders, and suggesting natural selection favors genome architectures that maximize fitness. But it is unclear whether rearranged genomes hinder adaptability: capacity to evolutionarily improve in a new environment. Negative-sense non-segmented RNA viruses (order *Mononegavirales*) have specific genome architecture: 3′ UTR – core protein genes – envelope protein genes – RNA-dependent RNA-polymerase gene – 5′ UTR. To test how genome architecture affects RNA virus evolution, we examined vesicular stomatitis virus (VSV) variants with the nucleocapsid (N) gene moved sequentially downstream in the genome. Because RNA polymerase stuttering in VSV replication causes greater mRNA production in upstream genes, N gene translocation toward the 5′ end leads to stepwise decreases in N transcription, viral replication and progeny production, and also impacts the activation of type 1 interferon mediated antiviral responses. We evolved VSV gene-order variants in two prostate cancer cell lines: LNCap cells deficient in innate immune response to viral infection, and PC-3 cells that mount an IFN stimulated anti-viral response to infection. We observed that gene order affects phenotypic adaptability (reproductive growth; viral suppression of immune function), especially on PC-3 cells that strongly select against virus infection. Overall, populations derived from the least-fit ancestor (most-altered N position architecture) adapted fastest, consistent with theory predicting populations with low initial fitness should improve faster in evolutionary time. Also, we observed correlated responses to selection, where viruses improved across both hosts, rather than suffer fitness trade-offs on unselected hosts. Whole genomics revealed multiple mutations in evolved variants, some of which were conserved across selective environments for a given gene order.

## Introduction

Gene order is a property of genetic architecture often conserved among closely related species, and mechanisms such as gene codon bias and physically interacting conserved gene pairs can cause altered gene order to decrease individual fitness (Dandekar et al., 1998). For viruses, which have significantly smaller genomes than most eukaryotes and prokaryotes, gene order can provide transcriptional control over viral protein production; thus, gene order is often conserved at the highest levels of virus classification, virus orders and families (Koonin et al., 1993; Conzelmann, 1998).

The order *Mononegavirales* contains non-segmented negative-sense RNA viruses, spanning five families of plant and animal viruses that include emerging pathogens such as Ebola virus, and Nipah virus (King et al., 2012). Genome architecture in the *Mononegavirales* is characterized as 3′ UTR – core protein genes – envelope protein genes – RNA-dependent RNA-polymerase gene – 5′ UTR. In the *Rhabdoviridae* virus family, this gene order determines the relative expression levels of each viral protein coded by the virus genome. Rhabdoviruses contain five canonical viral genes, with transcriptional stop and start sites between each gene (Lyles and Rupprecht, 2007). As the polymerase moves down the genome, it stutters at the intergenic regions, leading to polyadenylation and mRNA termination, and some proportion of the polymerase drops off at each intergenic site. Therefore, transcripts are expressed in a gradient, such that viral replication causes proteins at the 3′ proximal end of the viral genome to be produced at higher intracellular levels than those at the distal end (Lyles and Rupprecht, 2007).

Vesicular stomatitis virus (VSV; family *Rhabdoviridae*, genus *Vesiculovirus*) has a ∼11.2 kb genome encoding five proteins: the nucleocapsid (N) protein that encapsidates the genomic RNA, phosphoprotein (P) and large (L) protein which make up the polymerase, glycoprotein (G) involved in cell-surface binding, and matrix (M) protein important both for virion formation and inhibition of host antiviral gene expression (Rose and Whitt, 2001; Lyles and Rupprecht, 2007). The VSV genome is organized such that its genetic architecture consists of the five genes in the order: N-P-M-G-L. VSV is generally not pathogenic to humans, and grows easily in many cell culture systems and tissues owing to its broad tropism (Hastie et al., 2013). As with most RNA viruses, VSV has a high mutation rate, rapid generation time, and small genome size, making it a useful and efficient model for examining phenotypic and genotypic evolution (Moya et al., 2004). VSV has been engineered to express surface proteins from diverse viruses, including Ebola, HIV-1, and influenza A, which can stimulate protective immune responses against these pathogens (Bukreyev et al., 2006). Additionally, VSV has shown promise as a candidate for oncolytic virus therapy, as it replicates most efficiently in cells with diminished innate immunity such as cancer cells which often have impaired production of and/or response to interferon (Barber, 2005). Mutations that attenuate VSV growth in healthy immune competent cells can further enhance the safety of this anti-cancer therapy potential (Barber, 2005).

Taking advantage of a conserved sequence in the intergenic regions of the VSV genome, Wertz et al. (1998) engineered VSV variants with altered genetic architectures. While wild type VSV has the N gene located at the 3′ end of the genome (i.e., architecture N-P-M-G-L), the engineered strains have the N gene moved sequentially down the genome (Wertz et al., 1998). As the N gene is translocated toward the 5′ end of the genome, the production of VSV mRNA and subsequently N protein within the cell decreases while other genes’ production increases, impacting genomic RNA production, decreasing viral replication in cell culture, and attenuating virus infection in mice (Wertz et al., 1998).

The controlled attenuation of viral pathogenicity by gene translocation has been proposed as one method to make VSV more suitable as a platform for vaccine development. These and other mutations might limit the spread of VSV to neighboring cells with intact immune function (Ahmed et al., 2004; Lichty et al., 2004; Lam et al., 2005; Hastie and Grdzelishvili, 2012). Because the N gene location variants have gene orders that are not present in nature, these genotypes have not been subjected to the same selection pressures that have shaped wild type VSV. Importantly, the gene rearrangements described in the current study are unlikely to occur in nature, due to the replication mechanism and low recombination rate of viruses in Mononegavirales (Han and Worobey, 2011). In bacteriophage, genome rearrangement and subsequent evolution diminished the ability of the virus to adapt to new environments (Cecchini et al., 2013). We predicted that genomic reorganization in VSV should hinder the ability for the virus to adapt in a novel cellular environment, relative to wild type VSV.

We define adaptability as the fitness gained in a new environment, relative to the ancestral baseline fitness in this same novel environment. We hypothesized that as N is moved downstream in the genome, the relative reduction in N protein should cause fitness gains in evolved populations to rank according to genetic architecture: N1 (wild type) > N2 > N3 > N4. Importantly, this prediction assumes that N protein position (hence, abundance) should directly correlate with adaptive potential for VSV in a novel host environment. Alternatively, all rearranged genetic architectures may be equally hampered in adaptability on a novel host relative to wild type (N1 > N2= N3= N4), or genetic architecture may be irrelevant for adaptability (N1= N2= N3= N4). Finally, because novel genetic architectures appear to generally impair VSV fitness relative to wild type (Wertz et al., 1998), adaptive landscape theory predicts that a larger fraction of spontaneous mutations may be beneficial for the novel variants. A population which is reasonably well-adapted to a novel environment relies on mutations of small effect size as it evolves optimal performance (i.e., as it climbs a fitness peak in the adaptive landscape), whereas a poorly adapted population is likely to make larger ‘fitness jumps’ in an equivalent time, because it can improve through mutations of large effect size without overshooting the fitness-peak target (Fisher, 1930; see also Burch and Chao, 1999). Because the novel N variants may be further than the wild type from a fitness peak, they may experience stronger selection to climb the peak. By this logic, adaptability of rearranged viruses may exceed that of wild type (N1 < N2 ≤ N3 ≤ N4), because these variants could experience a relatively greater supply rate of large-effect beneficial mutations that speeds their adaptation (Crow and Kimura, 1970). Thus, we considered that the strength of selection imposed by the novel host environment could impact the relative adaptability of VSV populations founded by N-protein variants, suggesting the usefulness of comparing adaptability outcomes for N variants on novel hosts of differing selection strength.

To compare and contrast how gene order affects RNA virus adaptability, we used each N variant to found three replicate virus populations, in each of two novel host environments. Both novel hosts were derived from human prostate cancer, but differ in the strength of selection exerted against VSV. LNCaP cells have defects in multiple innate immune response pathways, making these cells highly permissive for infection and growth by VSV. In contrast, PC-3 cells have intact innate immunity and are responsive to interferon, making these cells relatively more restrictive for VSV growth (Ahmed et al., 2004). Thus, LNCaP cells constitute a relatively weak immune-selective environment for VSV, whereas PC-3 cells are expected to be a harsh environment that exerts strong selective pressure against the virus.

Here we experimentally evolved VSV for 30 consecutive daily passages (∼120 virus generations) to show that gene order affects RNA virus phenotypic adaptability (reproductive growth; viral suppression of immune function), especially on a novel host that exerts strong selection pressure against virus infection. We observed that the N4 strain ancestor was generally least fit among all N variants, but that populations derived from this strain experienced the greatest fitness change in the time allowed; this result was consistent with the most-altered N position genetic architecture being most favored to adapt. We further showed that correlated responses to selection allowed virus populations to improve simultaneously on both host types, rather than suffer performance trade-offs on hosts other than the evolutionary host (Turner and Elena, 2000; Novella et al., 2005; Remold et al., 2008). Further, the fitness gain by the N4 virus strain relative to its ancestor was associated with a greater rather than lower activation of host cellular responses, suggesting that increased fitness was not due to enhanced viral ability to block cellular immunity. Finally, we used whole genome sequencing of evolved populations to infer the genes/mutations associated with changes in phenotypic traits, and found multiple mutations in evolved variants, some of which were conserved across selective environments for a given gene order.

## Materials and Methods

### Cell Lines and Culture Conditions

Prostate cancer cell lines were obtained from American Type Culture Collection: PC-3 cells (ATCC #CRL-1435); LNCaP cells (ATCC #CRL-1740). These cells were grown in 37^∘^C incubators with 5% CO_2_ atmosphere, using RPMI 1640 media supplemented with 10% fetal bovine serum (FBS), and 1% mixture of penicillin, streptomycin, and L-glutamine. BHK-21 cells (kindly provided by Esteban Domingo, University of Madrid) were used for plaque assays, and were grown under the same conditions as above, except using Dulbecco’s modified Eagle’s minimal essential medium (DMEM) supplemented with 10% FBS, and 1% penicillin-streptomycin, l-glutamine. Low passage variants of cell lines (i.e., between 5 and 25 laboratory passages) were used for all experimental evolution and fitness assays.

### Strains

Nucleocapsid-position variants of VSV were kindly provided by Gail Wertz (School of Medicine at the University of Virginia). These variants have N translocated downstream in the genome, and are referred to by the position that N occupies: N1 is wild type with N gene occupying the first position in the genome, N2 has N gene in the second position and P gene in the first position, N3 has N gene in the third position following P and M genes, and N4 has N gene in the fourth position followed only by L gene. The genomes of these strains were sequenced prior to experimental evolution (GenBank #KP872861-KP872888), and they were identical to each other except for the N translocation, and the following point mutations (amino acid substitutions); N1: A274G (E92K) in G gene; N3: A600G (R179H) in N gene, and G4107A silent mutation in G gene; and N4: U9594G (Y1621D) in L gene.

### Plaque Assays

A serially diluted virus sample was added to a confluent monolayer of BHK-21 cells grown in 6-well plates, and incubated for 45 min at 37^∘^C with rocking every 15 min. Medium containing virus was removed, and replaced with a mixture of agar and 2x DMEM, and incubated for additional 24–30 h at 37^∘^C. Cells were then fixed with 10% formaldehyde, agar and medium was removed, and cells were stained with crystal violet to visualize plaques. Titers (plaque-forming units [PFUs] per mL) were estimated as averages of counts from dilutions in two separate wells.

### Experimental Evolution

Prior to experimental evolution, N1, N2, N3, and N4 virus strains were grown in BHK-21 cells (typical laboratory host) to generate ancestor stocks. For experimental evolution, each ancestor was used to infect confluent monolayers of PC-3 and LNCaP cells in 6-well plates, at multiplicity of infection (MOI) ≈ 0.1 PFU per cell. A total of 24 experimental populations were created (4 ancestors × 3 replicate populations × 2 hosts). Infection consisted of 200 μL virus solution added to the monolayer for 45 min at 37^∘^C; this inoculum was then removed and replaced with 1 mL of RPMI growth medium. Virus was harvested at 24 h post infection (hpi), and diluted 10^-3^ or 10^-4^, depending on the strain, to create MOI = 0.1 to initiate the next passage; a sample from each passage was stored at -80^∘^C. This process was repeated for a total of 30 passages, where it is estimated that VSV experiences four generations per passage under these culture conditions (Miralles et al., 2000); thus, duration of the experiment was roughly 120 generations of VSV evolution.

Every five passages, each population was titered via plaque assay as described above, to ensure proper dilution for MOI = 0.1. At passage 29, each population was passaged to three separate wells in 6-well plates and then combined at the end of the passage, to create a large stock of 200 μL aliquots for subsequent analysis. Each evolved endpoint (passage 30) population was assigned a strain name according to its founding ancestor (N1, N2, N3, N4), the host type experienced during experimental evolution (L30 for LNCaP 30 passages, P30 for PC-3 30 passages), and its replicate designation (A, B, C): N1L30A – N1L30C, N2L30A – N2L30C, N3L30A – N3L30C, N4L30A – N4L30C, N1P30A – N1P30C, N2P30A – N2P30C, N3P30A – N3P30C, N4P30A – N4P30C.

### Virus Growth (Relative Fitness) Assays

Each founding ancestor strain and evolved population was assayed for growth on LNCaP and on PC-3 cells, with threefold replication. A test virus was inoculated on a confluent cell monolayer in 6-well plates at MOI = 0.1, using culture conditions employed in the experimental evolution. A 50-μL virus sample of supernatant was obtained at 24 hpi. Samples were then serially diluted 10^-1^, and titered via plaque assays on BHK-21 cells to estimate population size.

To compare fitness across strains, each evolved population was inoculated into three wells of PC-3 and LNCap cells, and sampled at 24 hpi. The estimated titer of each evolved virus was divided by that produced by its ancestral virus (i.e., with the same genomic architecture), and this ratio was subtracted from 1.0 to estimate relative fitness (w), as described previously (Nougairede et al., 2013). Fitness of evolved strains was tested for each group on each cell type by one sample t test against the null hypothesis that means were equal to zero (GraphPad Prism 5).

### IFIT2GFP Reporter Cell Activity Assay

Immune activation of the ancestor and evolved strains was assayed using a stable PC-3 IFIT2GFP reporter cell line, described previously (Swick et al., 2014). PC-3 IFIT2 reporter cells were plated in 96 well plates and incubated for 24 h until they had reached 90% confluency (∼20,000 cells/well). Each ancestor and evolved population was diluted to proper concentration for MOI = 10 infection. Growth medium was removed from cells and replaced with RPMI plus 2% FBS containing Hoechst 33342 (Anaspec #83218) diluted 1:20,000 as a live-cell nuclear stain. Forty μl of virus inoculum was added to each well and remained on the cells over the course of the infection. At 20 hpi, cells were imaged by widefield microscopy (4x magnification) for GFP signal using Nikon TE Eclipse 300 fitted with an outer warming chamber set to 37^∘^C (InVivo Scientific) and a stage-top incubator chamber (Pathology Devices) set to 37^∘^C, 5%CO_2_, and 75% relative humidity. Fluorescence illumination was provided by a Chroma PhotoFluor and controlled with a Lambda 10-2 optical filter wheel. Images were acquired with a QImaging ExiAqua. Stage automation was carried out with a Prior ProScanII and all automated imaging was controlled with MetaMorph v.7.7.8. Both blue (Hoechst) and green (GFP) channels were imaged to identify cell nuclei and measure GFP intensity respectively. Each well was imaged in a 2 × 2 array with a 4X objective. JEXTools open source image processing software^1^ was used to identify cells based on their nuclei, measure GFP intensity in an ROI centered on the identified nucleus, and database all images and processing results. We developed an aggregate IFIT2 signal metric, defined as the number of GFP positive cells in the arrayed field multiplied by the mean intensity of positive cells, then normalized to the number of cells counted in the field. This method produced a better signal to noise ratio than other measures such as total field intensity or average cell intensity. The aggregate IFIT2 signal was used to compare evolved strains to their ancestor, and to compare between N rearrangements. The aggregate signal from 3 replicate wells was averaged for each of 3 biological replicates to minimize signal variability from experiment to experiment.

### Antiviral Secretion Assay

Separate wells of LNCaP and PC-3 cells were infected with each ancestor and evolved virus strain, in triplicate, and the infection was allowed to progress for 24 hpi. Supernatants containing secreted paracrine-signaling antivirals were collected and frozen for later assay. Secreted antivirals induced in either LNCaP or PC-3 cells by the ancestor and evolved viral strains were titered for antiviral paracrine-signaling activity. Upon thawing, active virus in the supernate samples was inactivated by exposure to 7000 J/m^2^ UVC irradiation with rocking over 20 min. Samples were then assayed for antiviral activity using published methods (Voigt et al., 2013). Briefly, samples were diluted serially 1:2, incubated for 24 h over A549 human lung epithelial cells (American Type Culture Collection strain #CCL-185) responsive to human antivirals, and challenged with a DsRed2-VSV reporter virus. After 24 h incubation, assay plates were scanned using a GE Typhoon FLA 9000 Biomolecular Imager at 555/580 nm. Mean fluorescent intensity of assay wells, in duplicate, was measured using JEXTools and used to calculate viral inhibitory activity present in the assayed antiviral secretions.

### Whole genome sequencing

RNA was extracted from 140 μL of viral stock using Qiagen QiAmp mini viral spin kit, and eluted in 60 μL water. Complementary DNA was prepared using superscript II reverse transcriptase. Virus was polymerase chain reaction (PCR) amplified using Taq polymerase (Promega), and VSV specific primers (Remold et al., 2008), using a touchdown PCR reaction from 60 to 48^∘^C. Amplicons were purified by gel extraction (Qiagen) or enzymatic inactivation (with Antarctic phosphatase and Exo1), and sequenced by Sanger sequencing at the DNA Analysis Facility on Science Hill at Yale University^2^. Chromatograms were loaded into CLC Main workbench 6, and checked for quality before assembling into contigs, with at least 2x coverage throughout the coding regions of the genome. Evolved strains were compared with ancestors of the same genomic architecture, to determine which changes occurred during experimental evolution. One consensus sequence was determined for each evolved strain.

## Results

### Fitness of Ancestral Viruses Containing Rearranged Genomes

**FIGURE 1:**
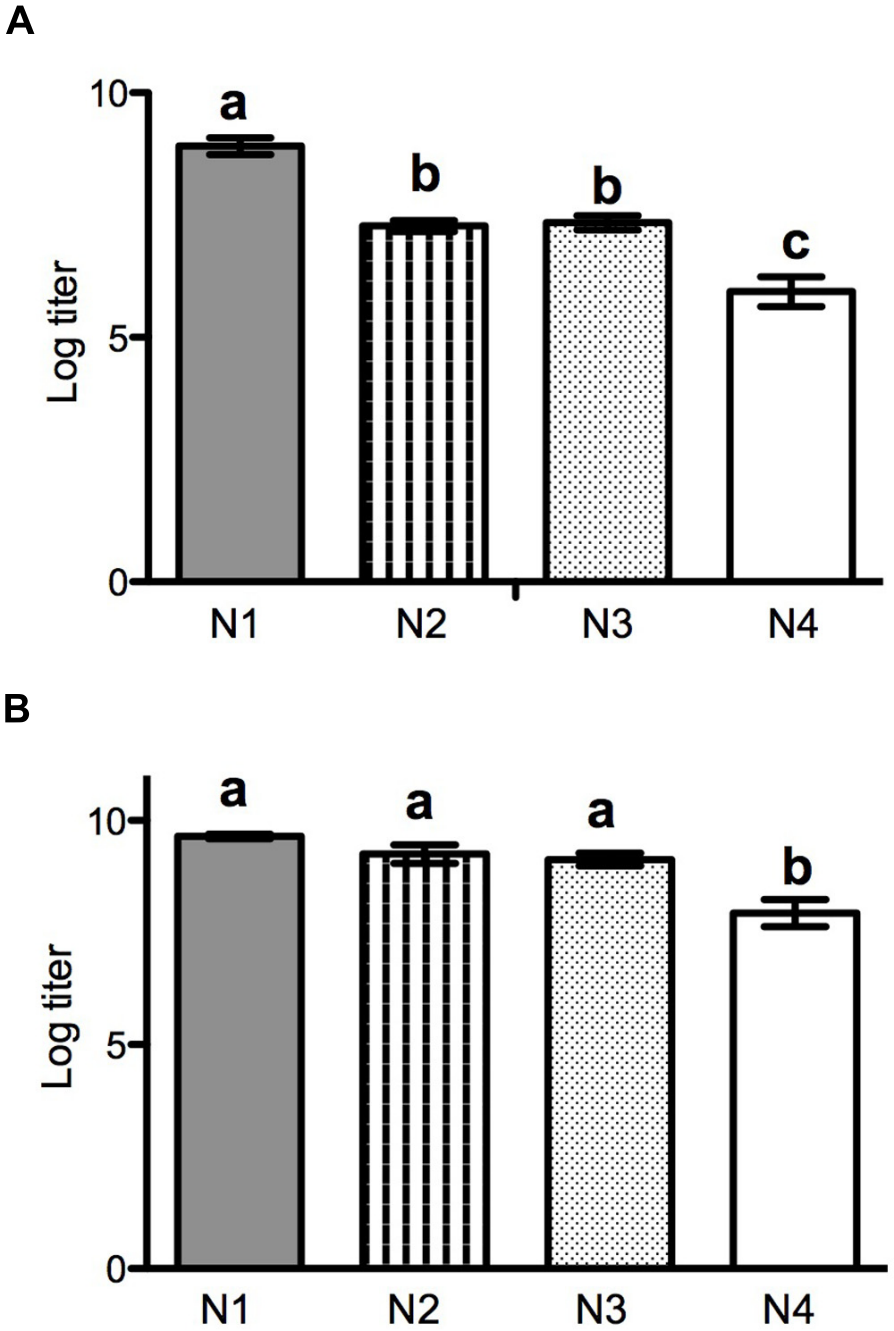
**Reproductive output after 24 h growth in PC-3 **(A)** or LNCap **(B)** cells for N1–N4 ancestral strains.** Output is shown as log titer produced (±SD, *n* = 3), as determined by plaque assay.

We assayed absolute fitness (total viable progeny [PFU] produced by 24 hpi) for each of the four ancestral VSV strains on both PC-3 and LNCaP cells, under culture conditions identical to those imposed during experimental evolution. Each ancestral strain was assayed with threefold repetition on each host. Results on PC-3 cells (**Figure 1A**) showed that the N1 (wild type VSV) ancestor was advantaged in growth relative to titers produced by the rearranged N genome variants (Tukey’s test: *p* < 0.005 for all three comparisons between N1 and the other variants). On PC-3 cells, the N2 and N3 ancestral strains did not differ in fitness (*p* > 0.05), whereas ancestor N4 was disadvantaged relative to all other variants (*p* < 0.005, all three comparisons). Results on LNCaP cells (**Figure 1B**) showed that this host type was generally more permissive for VSV reproduction, gauged by overall higher titers achieved by the viruses on this host and greater similarities in titers among the strains. On LNCaP cells, ancestral strains N1, N2, and N3 did not statistically differ in fitness (*p* > 0.05), but the N4 ancestor was relatively disadvantaged (*p* < 0.005, all three comparisons). Wertz et al. (1998) observed declines of roughly 0.5 log_10_ titer as N was moved downstream in the VSV genome, in assays using BHK-21 cells. In the current study, results on PC-3 cells echoed this earlier report, except that ancestors N2 and N3 tended to produce equivalent titers on the PC-3 host. In contrast, results on LNCap cells showed that titers were equivalent among three of the ancestral variants, with only the N4 strain showing relatively poor growth ability on this permissive host.

### Patterns of Absolute Fitness Changes and Correlated Improvements in Evolved Populations

To examine fitness gains of the 24 evolved VSV populations, we first estimated absolute fitness for each endpoint (generation 120) population with threefold repetition, on both PC-3 and LNCaP host cells. We then divided absolute fitness of an evolved strain by that of its particular ancestor (N1, N2, N3, or N4) measured on the same cell type (LNCaP, or PC-3). Finally, this value was subtracted from 1.0 to estimate fitness gained during the experimental evolution. Values significantly greater than 0.0 would indicate that the population gained in fitness relative to its own ancestor, whereas values less than 0.0 would indicate fitness was lost.

**FIGURE 2:**
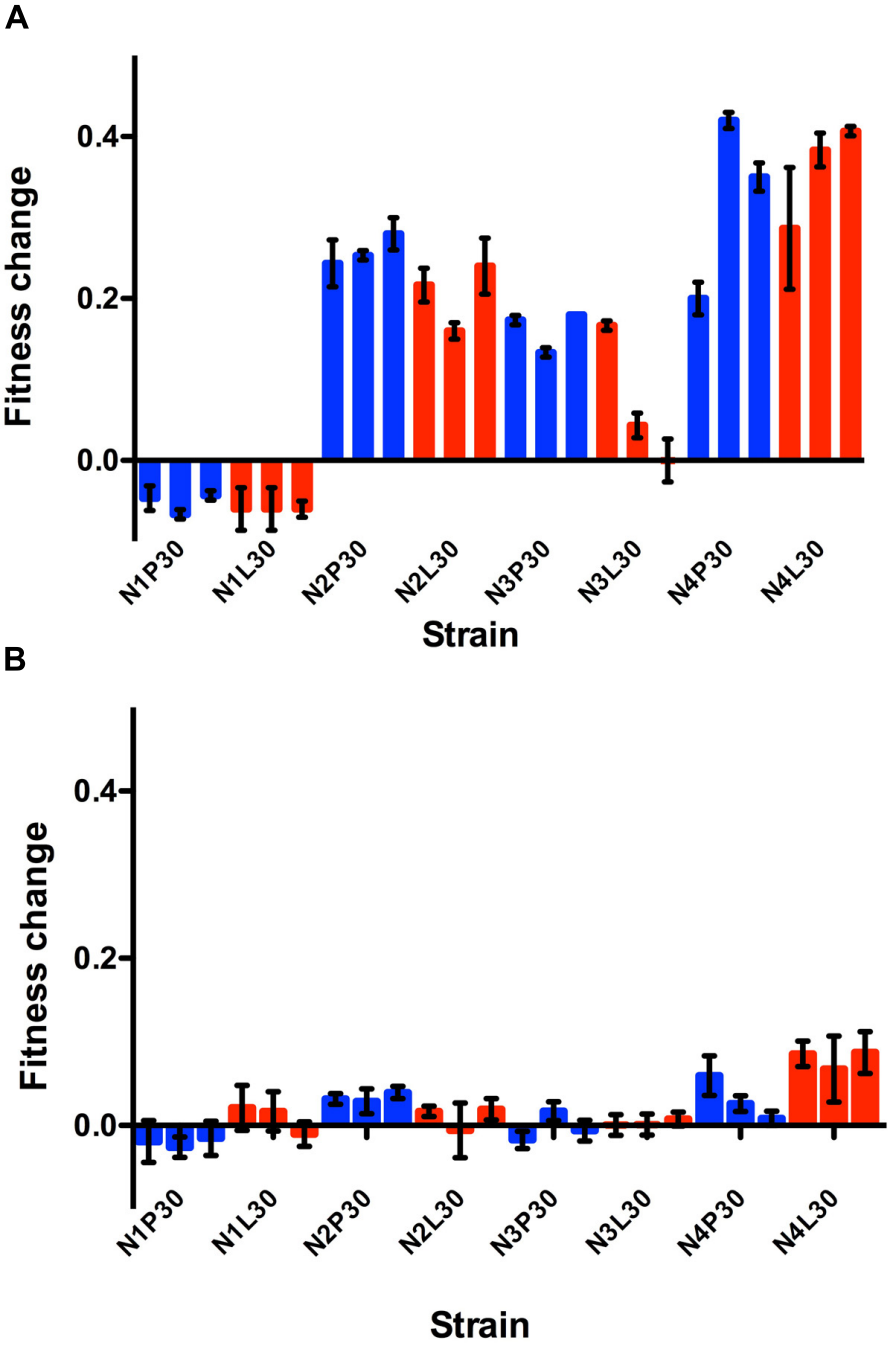
**Relative fitness is shown for each evolved replicate calculated after growth in PC-3 cells **(A)** or LNCaP cells **(B)**.** Viral productivity was measured at 24 h post infection in each cell type, as assayed by titration in BHK-21 cells. Fitness was calculated as 1-log (evolved virus production/ancestral virus production). Blue bars are for lineages passaged through PC-3 cells while red bars depict lineages passaged through LNCaP cells. Three replicates were infected and assayed for each evolved cell type, and the SE is shown.

Results (**Figures 2A,B**) showed variable absolute fitness gains among treatment populations, as well as among treatments. Overall, these fitness changes were more dramatic when measured using PC-3 cells (**Figure 2A**). Here, the vast majority of evolved populations that contained non-natural gene orders showed improved fitness relative to their respective ancestor (fitness change significantly greater than 0.0); the lone exception was population N3CL30, which evolved on LNCaP cells and showed fitness on PC-3 cells equivalent to its ancestor. However, this general trend was not true for the evolved populations founded by wild type N1 genome architecture; all of these populations showed fitness on PC-3 cells that was less than the ancestor’s. Interestingly, these overall trends tended to hold regardless of the selective host conditions imposed in experimental evolution. That is, absolute fitness increases and decreases on PC-3 cells tended to be similar for populations founded by a particular genetic architecture, regardless of whether selection had occurred on PC-3 versus LNCaP cells (**Figure 2A**; see further results and discussion below). We used two-way ANOVA to further examine effects of genetic architecture, host selective environment, and their interaction, on absolute fitness gains. Results showed that all three factors had significant effects (genetic architecture: *F* = 177.4, 3 df, *p* < 0.0001; selective host: *F* = 5.326, 1 df, *p* = 0.024; architecture^∗^host interaction: *F* = 4.713, 3 df, *p* = 0.005).

Changes in absolute fitness were more similar across the entire set of evolved populations when fitness was assayed on LNCaP cells (**Figure 2B**). Here, the observed mean changes were relatively modest overall, and often indicated that evolved performance on LNCaP cells was unchanged relative to the respective ancestor. Thus, in many instances the 120 generations of experimental evolution proved inconsequential for absolute fitness on LNCaP cells, including for some populations selected on this host. However, one overall trend revealed by the data was that N4-founded populations tended to improve the most, on average, during experimental evolution, on both PC-3 and on LNCaP cells. This trend was evidenced by the tendency for these N4 populations to improve greatly on PC-3 cells, and to improve more than their N1 through N3 counterpart populations on LNCaP cells even though changes on this host were modest (cf. **Figures 2A,B**). Again, two-way ANOVA showed that all three factors significantly affected absolute fitness gains on LNCaP cells (genetic architecture: *F* = 33.05, 3 df, *p* < 0.0001; selective host: *F* = 9.45, 1 df, *p* = 0.003; architecture^∗^host interaction: *F* = 10.47, 3 df, *p* < 0.0001).

**FIGURE 3:**
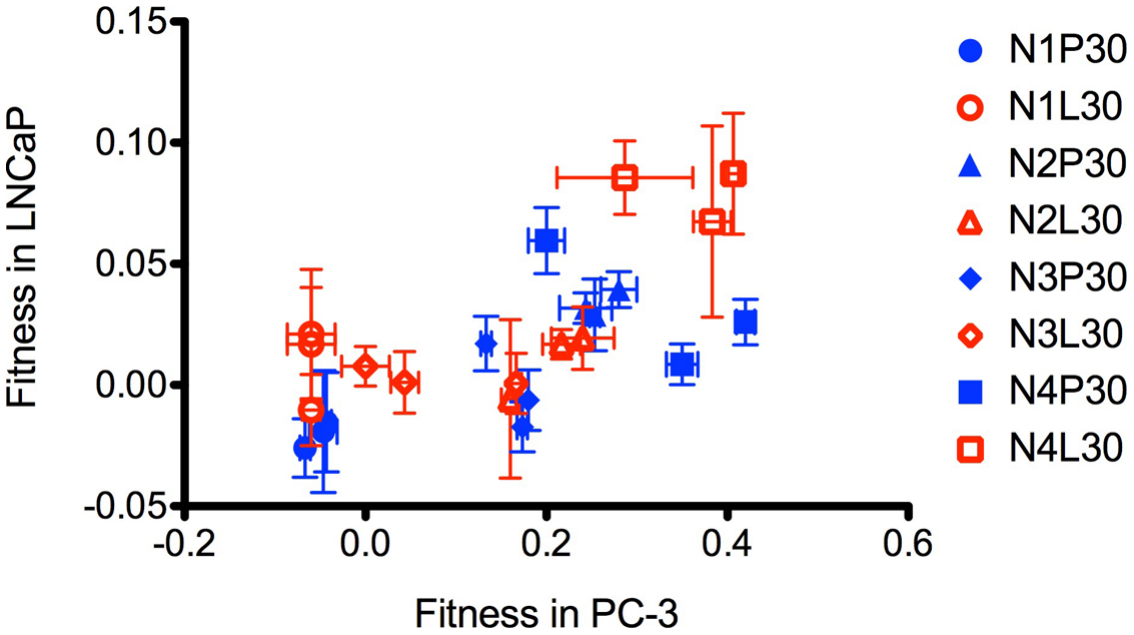
**Plot of fitness calculated relative to ancestor in PC-3 versus LNCaP cells.** Mean and SD of three replicates for each evolved strain are shown. LNCaP evolved strains are open shapes in red, PC-3 evolved strains are closed shapes in blue. N1 are circles, N2 are triangles, N3 are diamonds, and N4 are squares.

A virus population that adaptively improves in its selective environment may show reduced performance in an environment different than the selected environment (fitness trade-off). Alternatively, a virus population selected in one environment may also show improvement in other environments (correlated response to selection). To examine these possibilities, we plotted population data for mean changes in absolute fitness after evolution on PC-3 vs. LNCaP cells (see **Figures 2A,B**). These results (**Figure 3**) showed a positive correlation (*r*^2^ = 0.43, *F* = 16.61, *p* = 0.0005). This test confirmed that improvement in one selective environment tended to coincide with improvement in the other unselected environment, which is the expected trend for correlated fitness improvements. These results suggested that host cell origin (prostate cancer) and the general conditions employed for laboratory tissue culture were more important selective forces in our experiment than the degree of intact immunity (permissiveness) presented by one cell type versus the other.

### Activation of Innate Immune Response by Ancestral and Evolved Variants

The ability for the ancestral strains and evolved populations to suppress host innate immune response was assayed by a stable cell transcriptional reporter, and levels of secreted antiviral cytokines. Using a stable PC-3 fluorescent reporter cell line that contains a promoter reporter construct for the innate immunity gene IFIT2 controlling ZsGreen1 (GFP variant), we measured the capacity of each test virus to induce intracellular mechanisms of innate immunity (Swick et al., 2014). The IFIT2 promoter contains a prototypical interferon stimulated response element (ISRE) site and can be induced by exogenous interferons through Jak/Stat signaling; however, studies have shown that it can also be activated directly by intracellular viral replication processes (Elco et al., 2005).

**FIGURE 4:**
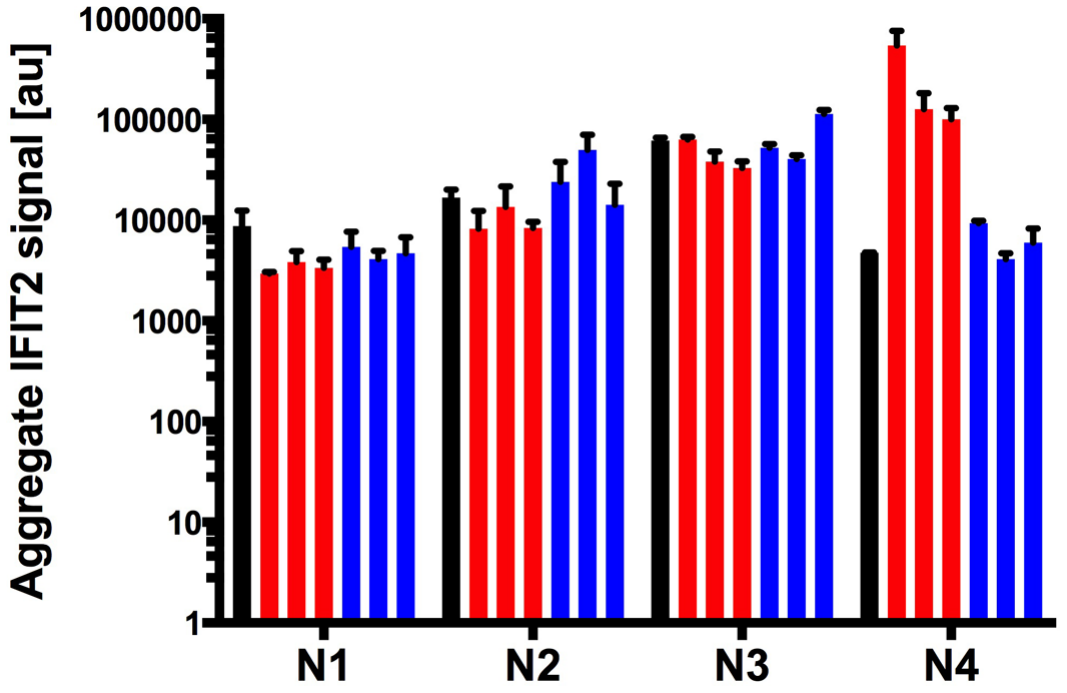
**Nucleocapsid rearrangement strains and their evolved variants display differing capacity to suppress host cell innate immunity.** Activation of host innate immunity was detected with a PC-3 IFIT2-ZsGreen stable reporter cell line. Induction of IFIT2 was measured as an aggregate signal metric defined in materials and methods. Ancestor populations for each N rearrangement are shown in the black bars, each of the three PC-3 and LNCaP evolved strains are shown in blue and red respectively. Error bars represent the SD of 3 biological replicates.

We infected the PC-3 reporter cells at MOI = 10 using each of the N rearranged ancestral and evolved variants, with threefold repetition, and we assayed relative levels of IFIT2 activation by fluorescence microscopy at 20 hpi. Surprisingly, all three of the evolved N4 populations on PC-3 cells exhibited more than 10-fold higher activation of the IFIT2 reporter relative to their ancestral strain (**Figure 4**, *p* < 0.02). Conversely, while not statistically significant, N1 populations evolved on PC-3 cells induced less activation of the IFIT2 reporter relative to activation by their ancestor (*p* < 0.1). Together, these results suggest that the IFIT2 reporter correlates more with the measure of infection–induced viral stress (or associated viral fitness) than activation of an immune-protected or non-permissive cell state; evolved reduced-fitness N1 strains (**Figure 4**) activate a reduced stress response (**Figure 2A**), while evolved higher-fitness N4 strains (**Figure 2A**) activate a more elevated stress response (**Figure 4**). For the N4 strains evolved on the LNCaP cells, IFIT2 activation was similar to the ancestor population.

Because populations evolved on LNCaP cells did not experience innate immune selection, we expected that these populations would show similar IFIT2 activation levels to their respective ancestor strains. In general, this prediction was upheld, according to the observed data (**Figure 4**).

### Stimulation of Paracrine-Signaling Antivirals by Ancestral and Evolved Variants

**FIGURE 5:**
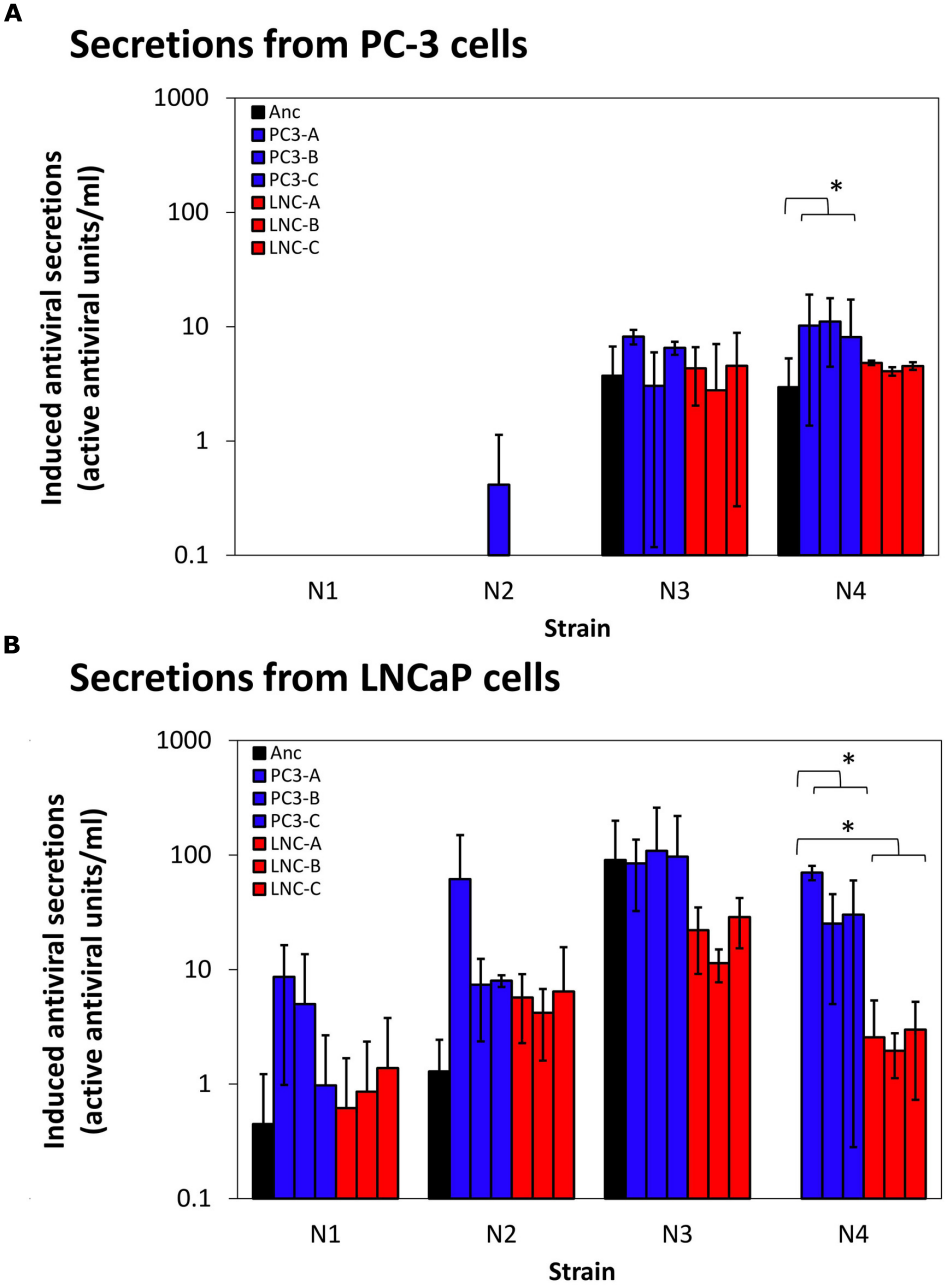
**Induction of auto/paracrine antiviral signaling by ancestor and evolved virus strains.** Antiviral paracrine signaling activity secreted into supernates above virus-infected cells [PC-3 cells, **(A)**, LNCaP cells, **(B)**] are shown. Data shown are mean and SD of three biological replicates for each evolved strain shown. Secreted activity due to ancestor strain infections are shown in blue, PC-3-evolved strains in reds, and LNCaP-evolved strains in greens. ^∗^*p* < 0.05.

PC-3 cells’ intact innate immune responses provide possible selection pressure against virus strains that induce large amounts of cellular paracrine-signaling antiviral molecules that activate antiviral states in neighboring cells. In contrast, LNCaP cells are deficient in their ability to respond to innate signaling molecules due to defects in the interferon signaling network that impair JAK/STAT signaling downstream of interferon secretion (Dunn et al., 2005). Therefore, it could be hypothesized that viral strains evolved on PC-3 cells would select for mutations that aid in blocking host immune protein production and/or secretion, a selection pressure not present for LNCaP evolution. To examine the immune-stimulating capabilities of each of the ancestral strains and evolved populations, we measured their stimulation of paracrine-signaling antiviral responses in both LNCaP and PC-3 cells. These measurements were obtained in triplicate for each test virus, on each cell type. Results are shown in **Figure 5**. On LNCaP cells (**Figure 5B**), we observed secreted measurable active antivirals in response to infection by most ancestral and evolved strains, with the sole exception of ancestor N4, which did not induce detectable antiviral secretions. Induction levels in response to evolved N1, N2, and N3 virus strains were not statistically different from ancestor induction levels. There was, however, a clear increase of antiviral stimulation for the N4 strains evolved on either PC-3 or LNCaP cells (*p* < 0.01 for each), corresponding to the strains with highest increased fitness.

Different patterns were observed in the assays conducted on PC-3 cells (**Figure 5A**). Here, the data showed that both ancestral and evolved N1- and N2-type strains were largely able to inhibit measurable antiviral secretions in PC-3 cells, whereas ancestors N3 and N4 induced successful PC-3 cell antiviral secretions. Induced PC-3 secretions by N3- and N4-type evolved virus strains echoed the results for their respective ancestral strains, showing negligible changes in immune stimulation over the ancestor strain (*p* > 0.3). The sole exception was the possible increase of stimulation levels by N4 PC-3-evolved virus strains (*p* = 0.049).

While pressure from immune responses was an intended part of viral selection pressure in our system, evolved virus strains generally showed no added inhibition of cellular antiviral responses above their ancestor strains. In fact, large increases in viral fitness (N4 rearrangement strains) resulted in stimulation of more, rather than less, antiviral secretion. Thus, we conclude that increases in viral fitness must be due to factors other than mutations that affect virus-immune interactions.

### Genomic Analysis of Evolved Variants

**FIGURE 6:**
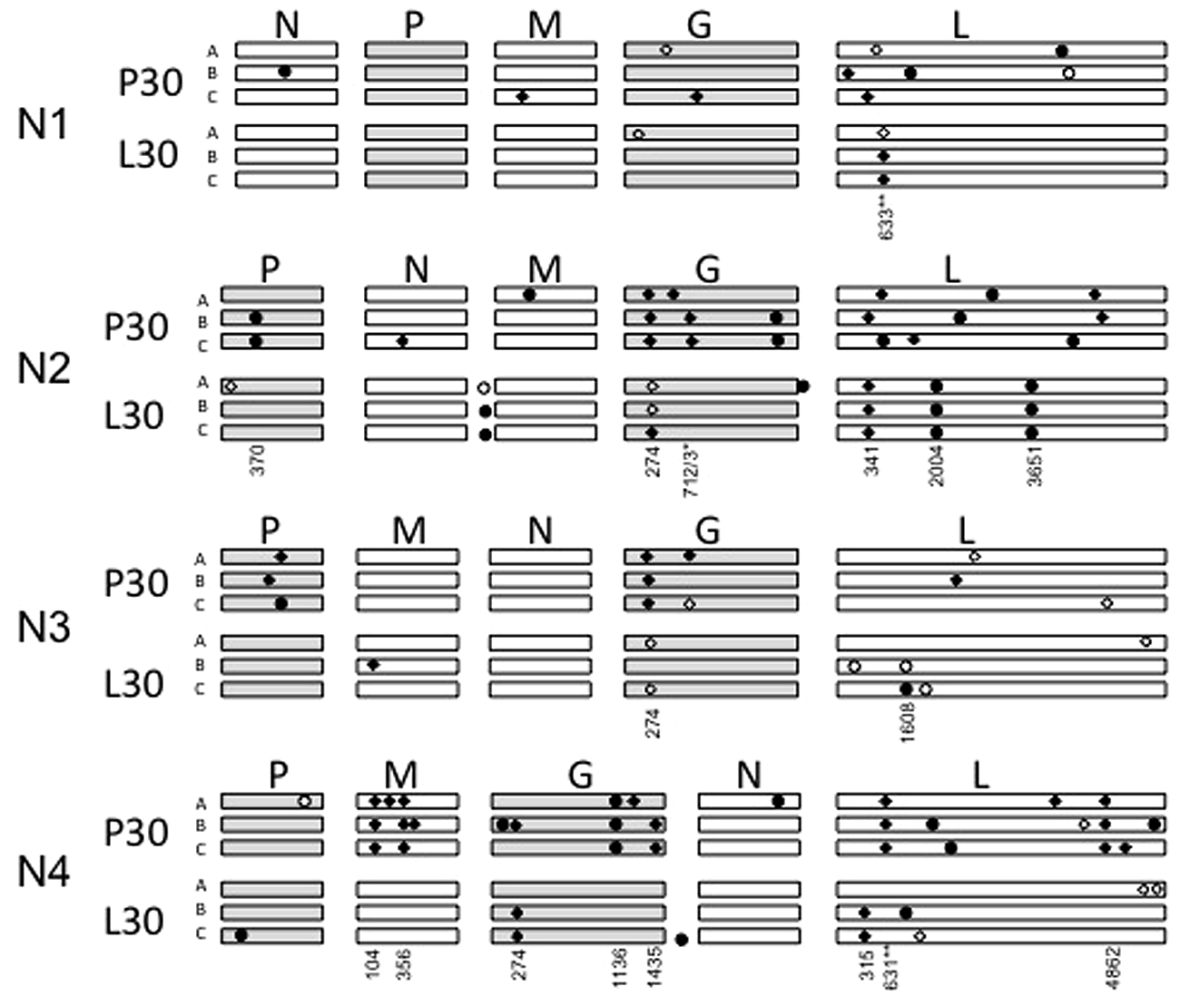
**Graphical depiction of mutations occurring in each evolved variant, not drawn to scale.** The approximate location of each mutation is depicted by circles (synonymous) or diamonds (non-synonymous), with polymorphic changes open circles or diamonds and fixed changes solid. The genomes are ordered from top to bottom by N translocation, which is also apparent in the given gene order. P30 means replicates passaged 30 times in PC-3 cells, L30 means replicates passaged 30 times in LNCaP cells. Numbers indicate the position of shared mutations (appearing in more than one lineage) relative to the start of each open reading frame.

We conducted whole-genome consensus sequencing of each evolved VSV population, to identify allele substitutions and polymorphic loci relative to the respective ancestral strain. Results in **Figure 6** show the locations of each change. **Table 1** provides further information on the nucleotide changes observed, and the predicted amino acid changes based on these mutations.

**Table 1:** Nucleotide and amino acid locations are given for each change to a coding region seen in the evolved lineages.

Gene order	Passage history		N	P	M	G	L	#/NS/P
N1	Pc-3 30	A				G649A/G (E217K/E)	G571A/G (R191G/R), G4122C (s)	3/2/2
	Pc-3 30	B	U660C (s)				A329G (Y110C), C1368T (s), U4201C/U (s)	4/1/1
	Pc-3 30	C			U281G (M94R)	G802A (E268K)	G463A (A155T)	3/3/0
	Lncap 30	A				A3080A/G (N25D/N)	U633G (F211L)	2/2/1
	Lncap 30	B					U633G (F211L)	1/1/0
	Lncap 30	C					U633G (F211L)	1/1/0
N2	Pc-3 30	A			G363U (s)	A274G (E92K), G580A (D194N)	A573C (R191S), G2886A(s), U4691C(I1564T)	6/4/0
	Pc-3 30	B		U370C (s)		A274G (E92K), C712A (Q238K), U1131C(s)	A341G(H114R), U2589C(s), U4721C(L1574S)	7/4/0
	Pc-3 30	C	G536A (R179H)	U370C (s)		A274G (E92K), A713G (Q238R), U1131C(s)	U631G (s), A3947G(D1316G), A4167G(s)	8/4/0
	Lncap 30	A		G4A/G (D2N/D)	T2242T/C (M mRNA)	A274G/A (E92K/E), A4696G	A341G(H114R), C2004G(s), U3651C(s)	7/3/3
	Lncap 30	B			T2242C (M mRNA)	A274G/A (E92K/E)	A341G(H114R), C2004G(s), U3651C(s)	5/2/1
	Lncap 30	C			T2242C (M mRNA)	A274G (E92K)	A341G(H114R), C2004G(s), U3651C(s)	5/2/0
N3	Pc-3 30	A		G481U/G (A161S/A)		A274G (E92K), C712A (Q238K)	U2550A/U (H850Q/H)	4/4/2
	Pc-3 30	B		G284A (Y95C)		A274G (E92K)	G2942A (R981K)	3/3/0
	Pc-3 30	C		A480G (s)		A274G (E92K), A713G/A (Q238R)	U4984C/U (H994Y/H)	4/3/2
	Lncap 30	A				A274G/A (E92K/E)	A4175G/C/A (Q1392R/P/Q)	2/2/2
	Lncap 30	B			G67A (A23T)		A234G/A (s), G1608A/G(s)	3/1/2
	Lncap 30	C				A274G/A (E92K/E)	G1608A(s), A1714G/A(s)	3/1/2
N4	Pc-3 30	A	A1053G (s)		A104G (Y35C), A155G (D52G), A356C (T119P)	A1136U (s)	U631C (F211L), G4862U(D1621Y), C3893U(A1297V)	8/6/0
	Pc-3 30	B			A104G (Y35C), A356C (T119P), C394A (H132N)	G123A (s), G274A (E92K), A1136U (s), G1186A(M362Y), U1435G (F479V)	U631C (F211L), G4862U(D1621Y), U1774C(s), U4661C/U(L1554S/L), A6015C (s)	13/9/1
	Pc-3 30	C		U726U/C (s)	A104G (Y35C), A356C (T119P)	A1136U (s), U1435G (F479V),	U631C (F211L), G4862U(D1621Y), G2175A (s), A5532T (L1844F)	9/6/1
	Lncap 30	A					A6247U/A(I2083F/I), C6274U/C (R2092W/R)	2/2/2
	Lncap 30	B				A274G (E92K)	U315G (D105E), C741A (s)	3/2/0
	Lncap 30	C		C91A (s)		A274G (E92K), G3195C (G mRNA)	U315G (D105E), G1448T/G (M483R/M)	5/3/1

*The ancestral nucleotide is shown, followed by the location relative to the start of the open reading frame for each protein coding region, and then the mutant allele detected in the evolved lineage. For non-synonymous changes, the predicted amino acid change is shown in parantheses with the same format while synonymous changes are indicated by (s). The last column shows the total number of mutations detected in genome, NS, the number of non-synonymous changes, and P, the number of polymorphic changes.*

Across the entire experiment, we identified a total of 111 new or polymorphic alleles in the genomes of the evolved VSV populations (**Figure 6**; **Table 1**). More mutations occurred for the populations evolved on PC-3 cells (72 mutations), compared to their counterparts evolved on LNCaP cells (39 mutations). This excess of mutations associated with PC-3 selection also held true for each paired set of populations founded by a particular N1–N4 ancestral variant. The greatest number of observed mutations occurred for N2- and N4-derived populations (38 and 40 mutations, respectively). More observed changes were fixed (78 sites) than were polymorphic (33 sites). In terms of predicted amino-acid changes, 71 observed changes were non-synonymous substitutions whereas the remaining 40 changes were synonymous substitutions.

Only four mutations were shared across multiple different N1–N4 genomic architectures. Substitution E92K in the VSV glycoprotein occurred in 20 of 24 lineages, including fixation of this substitution in the N1 ancestor prior to the start of experimental evolution. Substitution Q238R/K was shared between two replicate lineages of N2 and two lineages of N3 when evolved on PC-3 cells; F211L in the L protein coding region for all three N4 lineages evolved in PC-3 cells and all three N1 lineages evolved in LNCaP cells; and R191S, also in the L protein coding region, which is conferred by two distinct nucleotide changes G571A and A573C respectively for N1AP30 and N2AP30. Beyond these four mutations occurring in multiple different gene order variants, we observed an additional twelve mutations conserved between at least two replicate populations with the same gene order ancestor (**Table 1**). This overrepresentation of conserved changes occurring within a certain gene order and not experienced across a given cellular environment indicates N translocation may restrict the mutational landscape available to a given variant.

Several synonymous changes were conserved among populations, indicating selective pressure may occur for functions aside from coding of proteins. In the phosphoprotein gene, one synonymous change at nucleotide position 370 (U→C) was observed in two replicate lineages derived from N2 and passaged in PC-3 cells. In the glycoprotein gene, a conserved synonymous change occurred at nucleotide position 1136 (A1136U) in all three N4-derived replicates passaged in PC-3 cells. Other conserved mutations included three shared changes in all three lineages of N2 passaged in LNCaP cells; one in the non-coding region upstream of the M gene, and two in the G protein coding region: C2004G, U3651C. These shared synonymous changes could be compensatory changes, occurring after other coding changes have been selected in order to preserve RNA structure, or might have some other role in RNA replication or transcription.

We found unequal distribution of allelic changes across the five canonical VSV proteins, and observed that mutations were especially rare in the N coding region. This result was surprising, given that altered position of the N gene was the basis of our study. The nucleoprotein coding region had the fewest new alleles detected, with one allelic change detected for every 10,144 bases sequenced. In comparison, the phosphoprotein and polymerase coding genes (P and L) had relatively more changes, with allele substitutions every 2391 and 2865 sequenced nucleotides, respectively. The most frequently affected loci occurred in the M and G protein coding regions, where allelic changes were detected every 1181 and 1116 sequenced nucleotides.

## Discussion

Our study confirmed that gene order was consequential for the adaptability of an RNA virus in novel host environments consisting of innate immune competent or deficient cancer-derived cells. The ancestral fitness of the four N variants on immune-intact PC-3 cells and immune-deficient LNCaP cells “previewed” the outcome observed in absolute fitness gains, according to the prediction of strains with lower initial fitness tending to improve more in the evolutionary time allowed. In preliminary fitness assays on PC-3 cells, we observed that the N1 ancestral variant (wild type) grew best, the N2 and N3 variants were equally worse than N1, and N4 was the most impaired for growth.

### Impact of Selective Environment on Virus Fitness

Populations derived from N1-order strains did not gain fitness when evolved on either LNCaP or PC-3 cells. Lack of improved fitness in N1-order evolved strains has been found previously (Novella et al., 2005).

When evolved populations derived from N1-, N2-, and N3-order ancestors were assayed on PC-3 cells for fitness gains, we observed that N2- and N3-derived populations showed some improvement (although some variability was seen within and among treatments), and N4-founded populations gained the most in absolute fitness. Interestingly, this general trend held regardless of the selective environment experienced (PC-3 vs. LNCaP cells). Similarly, in preliminary assays we observed that N2 and N3 ancestors were equally fit on LNCaP cells, whereas the N4 ancestor had the worst performance. Again, this relative ranking “previewed” the outcome of experimental evolution because the N1 thru N3 evolved populations had minimal or no fitness gains on LNCaP cells, whereas all of the N4 evolved populations improved in absolute fitness.

Taken together, these data strongly suggested that the room for improvement of initially low-fitness strains determined their greater extent of fitness gained (adaptability) in the experimental evolution. This central finding of our study is in accord with classic predictions drawn from theoretical population genetics, concerning the relative speed of adaptation for populations of initially high versus low fitness in a novel selective environment (Fisher, 1930; Crow and Kimura, 1970). However, we note that this theory is primarily based on the presumed relative differences in abundance of beneficial mutations available to high fitness versus low fitness genotypes as they experience selection to improve in a new environment, where the genotypes are otherwise similar except for their distance from an adaptive peak on the fitness landscape. Our study extends the relevance of this population genetics framework to include genotypes that experience differing adaptability due to disparate genetic architectures, rather than due to genotypically different variants of the same genetic architecture.

### Gene Position Affects Molecular Evolution

As noted previously, decreasing intracellular abundance of N protein as N is translocated could hinder RNA virus adaptability. This prediction assumes that lesser adaptability should largely hinge on reductions in the intracellular prevalence of the ordinarily most abundant VSV protein, and ignores the possibility that changes in abundance of other proteins could instead drive adaptability. As N is translocated downstream, other genes are consequently moved upstream, causing these proteins to be present in overabundance compared to wild type levels. One could surmise that the enhanced adaptability of N4 derived populations has more to do with increased abundance of P, M, or G proteins that were moved upstream to accommodate the placement of N in the fourth position in the genome (i.e., P-M-G-N-L architecture, in contrast to wild type N-P-M-G-L or other novel architectures).

It is possible that the adaptive significance of these altered positions for P, M, and G proteins would lead to relatively greater numbers of fixed alleles in these VSV genes compared to the N gene. This expected correlation generally agrees with the observed prevalence of substitutions per base in our genomic dataset, because the N gene showed the fewest changes per base pair than any of the other VSV genes. However, all of the ancestor strains contained the L gene in the native wild type position, yet this gene also underwent greater changes per base than the N gene. This suggests that the effect of gene order on rate of allele substitution in VSV is not a simple relationship. Clearly, further work is required to examine this intriguing and unexpected result. In a computational study of VSV gene-order rearranged strains, Lim and Yin (2009) predicted the relative importance of gene position for each of the five VSV genes. Their results suggested that virus growth is most dependent on N and L gene positions, and less dependent on the relative genomic orders of the P, M, and G genes. Similarly, experimental studies of VSV strains that translocated the P, M, and G genes relative to one another but left the N and L genes in their wild type positions indicated that rearrangements of the P, M, and G genes alone affected viral growth only in relatively minor ways, including some strains that replicated slightly better than wild type (Novella et al., 2004a). This may help explain the propensity of the L gene to pick up mutations as a critical factor in viral replication. However, the relative lack of changes in the N protein then remains a mystery. The translocation of the N gene may tend to disrupt native epistatic interactions between VSV genes, and thus have led to selection for compensatory mutations in genes other than N to correct the problem. However, this is only one possible and highly speculative explanation. Future experiments could examine the possible role of gene order in dictating epistatic interactions between the VSV genes and the prospect that adaptation in a new host environment leads to novel sets of epistatically interacting beneficial mutations (e.g., see Remold et al., 2008).

Our genomics dataset also suggests that the number and type of underlying genetic changes differed greatly between evolved populations derived from different ancestral gene order variants. That is, translocation of the N gene could have led to gene-level convergence in observed allele substitutions for virus populations with different genetic architectures, challenged to adapt in identical novel host environments. Rather, we observed that the differing genetic architectures tended to further diverge at the genic level following experimental evolution, evidenced by different fixed substitutions and polymorphic sites in particular VSV genes. This result implied that a mutation that was selectively beneficial or neutral in one gene order variant was possibly deleterious in a different gene order variant. Rather than observing the same beneficial mutations regardless of VSV genomic order, we instead found that different mutations seemed to be favored according to gene order of the founding ancestor.

Although genetic exchange (recombination, reassortment) can be consequential for virus evolution (Perez-Losada et al., 2015), we note that recombination is either extremely rare or non-existent in VSV (Han and Worobey, 2011), and we detected no evidence that recombination led to restoration of wild type gene-order in populations founded by the N2–N4 variants. We cannot exclude the possibility that such recombination occurred in our study populations. However, recombination that reverted gene-order to wild type VSV would have conferred a growth benefit in the N2–N4 populations, presumably allowing these variants to reach high frequencies (or sweep to fixation). Such results were not observed.

### Fitness Increases do not Correlate with Increased Immune Evasion

As in all experimental evolution studies, details in experimental design will ultimately affect possible adaptive trajectories (Garland and Rose, 2009), and can help explain seemingly counterintuitive results. We observed that the N4-derived populations successfully adapted to PC-3 cells to a greater extent than their counterpart lineages. Thus, the robust activation of IFIT2 and secreted antiviral molecules by the N4-derived populations is seemingly at odds with their observed major fitness gains on this host. This result can perhaps be explained by the very poor initial growth of the N4 ancestor strain on PC-3 cells, which may be so weak that the virus does not induce a significant innate immune response. In contrast, the PC-3 evolved variants have amassed mutations that allowed greatly improved viral growth on PC-3 cells, in turn potentially increasing viral detection by the host and activation of antiviral responses. Alternatively, the relatively high MOI (MOI = 0.1) used during our experimental passaging may have allowed VSV replication to outpace the timing of host innate immune functional responses, preventing these responses from significantly impacting viral replication. When viruses such as VSV are grown in tissue culture, infections are usually initiated at low MOI on confluent (maximum density) host cell monolayers, such that the rapidly growing virus population destroys the available cells long before the next serial passage imposed at 24 h (Novella et al., 2004b). At our experimentally controlled MOI = 0.1, it is expected that a VSV population would be capable of undergoing two rounds of replication (i.e., two generations) within the initial 8–10 h of infection (Miralles et al., 2000). In contrast, antiviral secretion in PC-3 cells is not detectable until 8 h postinfection, even at high MOI (Voigt et al., 2013), and full IFIT2 activation in PC-3 cells requires at least 15 h. Thus the mutations in N4-derived populations that provided the major absolute fitness gains may also have increased immune activation, but such activation was too slow to negatively impact viral replication with the experimental protocol used in our experiment. That is, increases in absolute fitness were correlated with increased production of viral progeny, rather than with better modulation of the innate immune response within PC-3 cells.

This contention is further supported by the high frequency of matrix (M) protein mutations in the N4 PC-3 adapted strains. One of the main functions of the M protein is the suppression of host immune responses, and mutation of this protein, such as the well-described M51R variant, can abolish this suppressive function (Ahmed et al., 2003). From these initial results we would hypothesize that the accumulated M mutations in the N4 PC-3 evolved strains provided replication or other growth advantages, but also reduced immune suppression function. However, in the experimental design used in the passaging phase of the study such deleterious effects on fitness were potentially masked by the relatively high MOI. Notably, the N4 LNCaP adapted strains that did not induce increased IFIT2 activation lack any M protein mutations.

### Implications and Future Directions

In future work examining the gene order phenomenon it would be intriguing to consider the importance of transmission time (serial passage durations other than 24 h) in experimental evolution, as shown to be consequential for evolved VSV traits in other studies (Wasik et al., 2014). Additionally, follow-up work could examine the possible importance of MOI and its interactions with gene order in driving adaptability in the virus.

Our study suggests that rearranged gene order alone is insufficient as a strategy to permanently attenuate VSV, as virus populations with unnatural gene orders can successfully respond to strong selection that improves their growth despite the genome rearrangement. VSV strains with rearranged genomes are capable of infecting and destroying cells with impaired immunity, making these genotypes strong candidates for attacking certain cancer cells in oncolytic virus therapy. However, based on our findings, these strains experience relatively *stronger* selection to adapt the ability to infect and destroy normal cells in healthy tissue surrounding tumors. This bias in selective strengths suggests that anti-cancer therapy approaches that rely on viruses with altered gene order should prudently consider within-host selection as a possible disruptor of treatment goals. However, we note that our study is merely one initial step in examining effects of gene order on oncolytic virus therapy. We suggest that such therapies involving viruses should be considered through the lens of evolutionary medicine (Turner, 2013), because viruses are “self-amplifying drugs” that have the potential to evolve during the course of treatment. We do not suggest that such promising therapies should be abandoned; rather, we caution that development of any therapy should include consideration of its proximate and ultimate evolutionary implications. Subsequent work following the current study could use reverse genetics to confirm the functional role of allele substitutions that appear to promote improved growth of gene-order variants on cancer versus ordinary cells. Additionally, further work could incorporate a mathematical modeling approach in which the adaptive dynamics of the virus population are predicted in heterogeneous environments containing mixtures of cancerous and ordinary tissues. It would also be important to examine these effects of gene order on VSV adaptability in an environment that more closely mimics that of a human cancer patient, such as in mouse models of human cancers considered oncolytic therapy candidates.

## Author Contributions

KP: main design and execution of evolution experiment; genome sequencing and analysis; writing and editing of manuscript. EV: design of experiments; execution of antiviral secretion studies; writing and editing of manuscript. AS: design, execution, analysis, and summary of IFIT2 reporter cell experiments; editing and commenting on manuscript. VM: genome sequencing and analysis; feedback on manuscript. CT: interpretation of antiviral interaction and other data; feedback on manuscript. JY: design and interpretation of experiments; writing and editing of manuscript. PT: design and interpretation of experiments; writing and editing of manuscript.

## Conflict of Interest Statement

The authors declare that the research was conducted in the absence of any commercial or financial relationships that could be construed as a potential conflict of interest.

## Footnotes

1 http://sourceforge.net/projects/jextools/

2 http://dna-analysis.research.yale.edu/
